# Race and Ethnicity in Facebook Images and Text: Thematic Analysis

**DOI:** 10.2196/62713

**Published:** 2025-09-04

**Authors:** Shaniece Criss, Sarah M Gonzales, Heran Mane, Katrina Makres, Dalmondeh D Nayreau, Vaishnavi Bharadwaj, Hannah G Kim, Thu T Nguyen

**Affiliations:** 1 Department of Health Sciences Furman University Greenville, SC United States; 2 School of Public Health, Epidemiology and Biostatistics University of Maryland College Park, MD United States

**Keywords:** Facebook, race, ethnicity, racism, antiracism, cultural pride, solidarity, qualitative research

## Abstract

**Background:**

Social media platforms, such as Facebook, provide a dynamic public space where users of various racial and ethnic backgrounds share content related to identity, politics, and other social issues. These platforms allow racially minoritized groups to both challenge racial silencing and express cultural pride. At the same time, they expose users to racism and stereotypes that can negatively affect their mental and physical health through psychosocial stress. Given the rise of multimodal communication, it is essential to study both images and text to fully understand how race and ethnicity are discussed in digital spaces.

**Objective:**

This exploratory, descriptive study aimed to investigate how people discuss race and ethnicity on Facebook and specifically examine themes related to cultural pride, solidarity, racism, antiracism, and politics using qualitative content analysis of race- and ethnicity-related Facebook posts with images and text. These themes reflect how individuals construct identity, engage with other social identities, and navigate sociopolitical discourse in digital spaces.

**Methods:**

We conducted a qualitative content analysis using a hybrid inductive-deductive approach. A total of 500 multimodal Facebook posts were randomly sampled using CrowdTangle, with 100 posts from each year between 2019 and 2023. Each post included both image and text and contained at least 1 race- or ethnicity-related keyword. Posts were uploaded to GitHub for storage and to Label Studio for coding. An iteratively developed codebook guided the analysis, focusing on representations of race and ethnicity, the continuum of race-related discourse, and topical content. All posts were double coded until an 80% interrater agreement was reached. The remaining discrepancies were resolved through coder consensus to ensure reliability and consistency. Themes were solidified through thematic analysis.

**Results:**

Across 500 Facebook posts from 2019 to 2023, nearly one-third lacked clear racial specificity, with 19.8% (99/500) unrelated to race and 11.2% (56/500) mentioning no specific racial or ethnic group. Among the identified groups, Hispanic, multiracial, and immigrant communities were the most frequently referenced. Common themes included US politics, cultural pride, racism and stereotypes, and antiracism. Political content was the most crosscutting theme, while cultural pride and racism-related discourse varied by group. Antiracism posts reflected the national response to racial justice movements. These findings highlight the nuanced and evolving nature of race-related discourse on social media.

**Conclusions:**

It can be complicated to interpret image-based posts because of the subtle ways in which an image may reference race and ethnicity but does not explicitly mention it, or when there is a contradiction in the ideas portrayed in the image versus the text. Decoding this process on Facebook can help researchers boost the positive impacts and reduce the harmful effects of racism on social media.

## Introduction

### Race and Social Media

A total of 68% of adults in the United States reported that they have used Facebook at some point, with the racial and ethnic breakdown of users being 69% White, 67% Asian, 66% Hispanic, and 64% Black [1]. Facebook activity varies by race, with racially minoritized groups practicing more content creation and engagement related to race and ethnicity compared to White users and engaging more in the explicit self-presentation of racial and ethnic identities as salient [2-5]. This pattern of online content engagement creates resistance to the racial silencing of marginalized groups by dominant color-blind ideologies of broader society because racially minoritized groups can actively lead or participate in race- and ethnicity-related discourse [4,5].

However, while social media can provide opportunities to resist racial silencing and promote the voices of marginalized groups, it can also be used to propagate harmful and racist ideologies. Racism and the resulting stress for marginalized groups operate on intrapersonal, interpersonal, and organizational levels, and interpersonal racism can be disseminated through and observed on social media [6]. Vicarious racism, or awareness of racism against one’s friends, family, or racial group, creates stress and negatively impacts both physical and mental health outcomes [6,7]. Vicarious racism can also be mediated by social media. Racism has profound effects on the health outcomes of marginalized groups and is not only thought to correlate with stress and poorer health outcomes but is also thought to be a causal factor.

Engaging with race- and ethnicity-related content on social network sites is influenced by the anonymity or nonanonymity of the site, with Facebook predominantly being the latter [8]. The anonymity of social network sites allows users to more openly perpetuate hostility and negative impulses, whereas users of nonanonymous sites present more socially desirable traits of themselves [5,8]. Facebook’s nonanonymous status and the representation of racially minoritized groups on the platform provide support for the spiral of silence theory, which suggests that when faced with increasing social pressures, individuals with unpopular perspectives withhold their opinions (eg, racist ideals) due to fear of social isolation [8]. However, recent research suggests the spiral of silence theory is weakened by social media because nonanonymous Facebook users can engage in subtle or covert racism instead of overt racism in order to minimize social isolation [6,8]. It is also important to note that social media users are exposed to a multitude of content, especially within online echo chambers, which may disrupt user perception of what is considered popular or unpopular. User disorientation further weakens the spiral of silence theory and the formation of extreme views regarding race online may influence offline perceptions of racism. The racial formation theory holds that racial categories can be created, destroyed, and transformed by society; hence, social media posts that reinforce racist or stereotypical ideologies can spread a negative racial concept among social media users [9,10]. Both racial formation theory and the subversion of the spiral of silence theory guided the research team to further investigate racism as a major content theme.

Social media users are encouraged to share unpopular opinions when they perceive that their opinions are correct, believe that the topic of engagement is of heightened importance, or hold a particular personal concern, regardless of social pressures [8]. As a result, while racist discourse may still be prominent on social media, there remains a high potential to counter hateful content through collective calibration; that is, norm setting and policing harmful content [6,8]. Finally, the nature of social media networks, as characterized by racial homophily and a higher percentage of weak or strong ties, also has implications for engagement with ethno-racial content [2]. Racially minoritized groups report having weaker ties, usually for building social capital, which can lead to less intense race engagement. White individuals, by contrast, report having stronger ties, which means ethno-racial content creation or engagement happens within a reference group with similar values, perspectives, and a sense of solidarity [2,11]. There is a gap in the literature regarding the potential use of social media platforms to form stronger ties within minoritized groups over time, such as the cultivation of a sense of solidarity, pride, or other common values.

### Images in Social Media Analysis

Although images and image-based content on social media platforms circulate routinely, their contribution to social media communication can extend beyond entertainment. Expression of personal opinion, community engagement, and support for public causes are increasingly seen in image-based content, to the extent that videos and images are as common as written posts on social media [12,13]. The uses of social media, heightened by the use of images, contribute to the creation of collective group identity as well as group solidarity [14,15]. This makes social media platforms, such as Facebook, X (formerly known as Twitter), and Instagram, powerful settings for content creation and the dissemination of messages on diverse subjects and groups of people.

Image-based content on Facebook, such as memes, may contain implicit and explicit messages of racism or hate, frequently influenced by stereotypes of marginalized groups. However, memes often convey negative sentiments toward marginalized groups in a mitigated or ironic manner, often incorporating sarcasm [13,16]. Humor framing helps mask their hateful intent and avoid detection by content moderators, which aids their widespread distribution [17,18]. Unfortunately, the difficulty in detecting racism in image-based social media posts can perpetuate stereotypes and intolerance. For instance, Windisch and Simi [15] found that sharing humorous images with undertones of racism facilitated a sense of solidarity among White supremacists in a more entertaining manner than hate speeches or publications. The use of irony and humor in memes can allow social media users to express hateful sentiments toward marginalized groups without risking major repercussions. Therefore, image-based content can reveal common racial sentiments and public attitudes about stereotypes, which are multifaceted [19]. In addition, the viewer’s interpretation of posts is subjective and based on their personal identities, making users’ intentions difficult to identify.

### Gaps and Study Aim

There is a need to clearly describe qualitative content themes in Facebook posts regarding race and ethnicity [20,21]. Conventional studies and content moderation have often relied on text-based content. However, the advent of social media platforms rich with multimodal content has underscored the need to look beyond text and incorporate other modalities, such as images, to capture a more comprehensive and nuanced understanding of trends and sentiments expressed on these platforms. Using qualitative content analysis of race- and ethnicity-related Facebook posts with images and text, this exploratory, descriptive study seeks to investigate how people discuss race and ethnicity on Facebook and specifically examine themes related to cultural pride, solidarity, racism and antiracism, and politics. These themes reflect how individuals construct identity, engage with other social identities, and navigate sociopolitical discourse in digital spaces.

## Methods

### Sample

The relevant data include Facebook posts with images and text. For the remainder of the paper, the data will be referred to as posts. The study sample consisted of posts with terms related to race and ethnicity posted from 2019 to 2023. The data were collected using CrowdTangle, a public insight tool by Meta [22,23]. CrowdTangle tracks publicly available social media posts made by pages and groups covering 2 million public Instagram accounts, about 20 thousand of the most active subreddits, and over 7 million pages and groups [22,23]. For this study, a sample of 100 randomly selected Facebook posts per year that contain 1 or more race-related key terms was analyzed. Beyond setting the date range, the collection was further refined by restricting the data to posts in English, from Facebook pages that were primarily managed in the United States, and where at least 1 keyword from a race- and ethnicity-related keyword list (Multimedia Appendix 1) appeared in the post text or corresponding image text. To generate the keyword list, the research team used an online database of racial slurs [24], as well as a keyword list from a study that evaluated race-related online discourse [25] and categorized it by racial and ethnic groups. For this study, posts were randomly selected, specifically 100 Facebook posts per year that had images and that contained 1 or more race-related key terms.

### Data Management

For each year, a file was created containing the image URLs and their corresponding texts through GitHub. Subsequently, the data were uploaded to Label Studio Enterprise, where a project was created for each year, and the files containing the images, URLs, and texts were uploaded. The Label Studio platform offers a customizable and user-friendly interface that enables annotators to code efficiently and conduct interrater reliability through percent agreement [26].

### Codebook Development

The initial draft of the codebook was informed by the literature reviewed in the introduction, which highlights key frameworks and findings related to race and racism in digital and social media contexts. Drawing from this foundational literature, we identified initial constructs and categories for analysis, including racism, antiracism, solidarity, and various racial and ethnic group representations. The research team used a hybrid inductive-deductive approach by applying concepts from the literature while also refining categories through close reading and iterative coding of posts from our sample. The research team labeled 50 posts together and made revisions based on the piloted posts. Next, 2 researchers coded 100 posts, and the team met to make revisions to the codebook. The final codebook consisted of 3 major coding dimensions, as presented in Textbox 1.

**Coding dimensions.**

**Race and ethnicity referenced**

- Arab, Middle Eastern, Muslim, Asian, Black, Hispanic, indigenous, Immigrants, Jewish, White, discussed Black and White, multiple races, or no race specified.
- Each post was categorized based on which racial or ethnic groups were explicitly or implicitly referenced in either the image or the accompanying text. These categories were selected to capture the diverse ways in which race and ethnicity appeared across the dataset, whether through direct identification, symbolic representation, or discursive framing. Posts that mentioned more than one racial group were coded as “discussed Black and White” or “multiple races, multiethnic, or mixed,” depending on the content. Posts that did not refer to any specific group but still addressed racial issues were coded as “no specific race mentioned.” This approach allowed for both specificity in capturing group-related discourse and flexibility in acknowledging the complex, intersectional nature of many posts.

**Continuum of race-related discourse**

- Racist, antiracist, solidarity, and not racist.
- Posts were evaluated by the continuum of race-related discussions with classifications of racist posts, antiracism, solidarity, and posts that were not racist. *Racist* posts included aspects of discrimination, stereotyping, criticism of critical race theory, and slavery. The *antiracist* posts featured condemning racial discrimination, antihate, pointing out hypocrisy, and acknowledging White privilege. The *solidarity* response option included posts with aspects of cultural pride, representation, or unifying events. *Not racist* posts had no obvious connection to racism.

**Topic of the post**

- For example, cultural pride, immigration, religion, US politics, military, food, daily life, racism, and antiracism.
- There were a multitude of topics. *Cultural pride* consisted of positive cultural sentiments, including promoting civil rights and attending events. The *immigration* code was assigned to posts related to residents of other countries entering the United States, with documentation and without documentation. *Military* posts related to the US military, veterans, or military history. *Religion* referred to posts about beliefs, practices, and communities associated with religion, spirituality, and prayers. *US politics* included posts about national politics and politicians (eg, Kamala Harris, Ketanji Jackson-Brown, and Donald Trump), as well as local government and policies. Posts were coded as *global politics* if they mentioned foreign relations or politics in other countries. Posts about *food* included advertisements and admiration of food. *Racism* referred to posts that discussed discrimination, stereotypes, and offenses related to race and ethnicity. *Antiracism* referred to posts that focused on denouncing racist ideologies and fighting against inequitable treatment.

### Data Coding and Analysis

Extensive multisession discussions about the posts were held. All posts were double coded within Label Studio [26], and the team met to ensure that at least 80% interrater reliability was reached by calculating the percent agreement. After the initial coding process was complete, 2 coders met to reach a 100% consensus within the entire dataset. Once the complete dataset had the same coding designation, a thematic analysis was conducted [27]. Each researcher individually identified themes in the data, and as a group, solidified the final themes in the data, using the various perspectives of the different racial backgrounds and life experiences of the study team.

### Ethical Considerations

This study was determined not to be human participant research by the University of Maryland, College Park institutional review board (2072551-1). In addition, the social media posts were anonymized, upholding user privacy.

## Results

Table 1 provides count data on the frequencies of posts mentioning various racial and ethnic categories in 2019, 2020, 2021, 2022, and 2023 in the sample. Table 1 shows the total percentage of the 500 posts categorized by racial and ethnic groups over the 5-year period. Notably, the 2 most frequent classifications were posts coded as “not relevant” to race or ethnicity, which made up 19.8% (99/500), and posts that referenced no specific racial or ethnic group, accounting for 11.2% (56/500). These 2 categories together comprised nearly one-third of the dataset, highlighting the prevalence of generalized or ambiguous racial discourse in the sample. Among posts with identifiable racial or ethnic references, the most frequently mentioned groups were Hispanic (n=54, 10.8%), multiple races, multiethnic, or mixed (n=53, 10.6%), and immigrants (n=48, 9.6%). Posts referencing Black individuals or communities accounted for 9% (n=45) of the posts, followed by Asian (n=38, 7.6%) and Arab, Middle Eastern, and Muslim groups (n=32, 6.4%). Less commonly represented groups included White (n=26, 5.2%), Jewish (n=20, 4%), and Indigenous or Native American (n=10, 2%). Posts that discussed both Black and White racial groups simultaneously made up 3.8% (n=19) of the data. Race-related language was present in a significant number of public Facebook posts. Explicit identification of racial or ethnic groups was inconsistent, with many posts either lacking specific group references or being tangentially related to race.

**Table 1:** Count and percentage sample of Facebook posts referencing different race and ethnicity categories by year (2019-2023).

Race and ethnicity category	2019 (n=100), n	2020 (n=100), n	2021 (n=100), n	2022 (n=100), n	2023 (n=100), n	Over the 5-year period (N=500), n (%)
Arab, Middle Eastern, and Muslim	2	5	17	5	3	32 (6.4)
Asian	7	8	5	7	11	38 (7.6)
Black	6	11	9	9	10	45 (9)
Hispanic	7	15	11	14	7	54 (10.8)
Immigrants	16	8	10	6	8	48 (9.6)
Indigenous or Native American	1	2	2	1	4	10 (2)
Jewish	7	2	1	3	7	20 (4)
White	4	3	6	5	8	26 (5.2)
Discussed Black and White	5	4	5	4	1	19 (3.8)
Discussed multiple races (multiethnic or mixed)	16	16	8	6	7	53 (10.6)
No specific race mentioned	13	11	11	11	10	56 (11.2)
Not relevant	16	15	15	29	24	99 (19.8)

Table 2 presents common content themes across racial and ethnic categories, reflecting the underlying narratives, sentiments, and issues most frequently associated with each group across the 2019 to 2023 sample. These themes emerged from the topic codes in the codebook and were shaped through an interpretive analysis of the narrative patterns within each racial and ethnic group. US politics was the most prevalent crosscutting theme, appearing in posts associated with 7 (64%) of the 11 racial and ethnic categories: Arab, Middle Eastern, Muslim, Black, Hispanic, immigrants, White, discussed Black and White, and no specific race mentioned. Political content typically referenced elections, immigration policy, public figures, and social justice debates, often with explicit or implied racial framing. Cultural pride emerged as a prominent theme in 4 categories: Asian, Black, Hispanic, and Indigenous or Native American. These posts frequently celebrated cultural heritage, community events, and milestones in representation, such as historic appointments or honors. Posts highlighting racism and stereotypes were especially prominent in content referencing Black, Hispanic, and multiracial identities, often in the context of public discourse on systemic racism, xenophobia, and media portrayals. These posts spiked during moments of national attention to police violence, border policies, and racialized political rhetoric. The theme of antiracism appeared distinctly in posts referencing Black individuals, discussions involving both Black and White groups, and posts where no specific race was mentioned. These posts included calls to action, personal reflections, and educational content, with a notable peak in frequency during the summer of 2020, coinciding with widespread mobilizations following the killing of George Floyd. These patterns display how racial and ethnic discourse on Facebook is shaped by both identity-specific issues and broader societal events that cut across groups.

**Table 2:** Common content themes by race and ethnicity category in the sample (2019 to 2023)^a^.

Race and ethnicity category	Over the 5-year period (N=500), n (%)	Common content themes (2019-2023)
Arab, Middle Eastern, and Muslim	32 (6.4)	US military deaths in Afghanistan, religion, and hijabs
Asian	38 (7.6)	Cultural pride, food, COVID-19, and stereotypes
Black	45 (9)	Racism, antiracism, US politics, cultural pride, Black history, Kamala Harris, and Ketanji Brown Jackson
Hispanic	54 (10.8)	Food, US politics, United States-Mexico border, immigration, stereotypes, Donald Trump, and cultural pride
Immigrants	48 (9.6)	US politics, politics for and against immigration, refugee and immigrant crises, and anti-immigrant sentiments
Indigenous or Native American	10 (2)	Cultural pride, Native American proverbs and blessings, and events and celebrations
Jewish	20 (4)	Solidarity—Christians and Jews and support for Jewish people
White	26 (5.2)	US politics, association with the United States. Republican party, antiracism, daily life, humor, and sarcasm—“redneck”
Discussed Black and White	19 (3.8)	US politics, antiracism, and addressing historical and current racism—global and the United States
Discussed multiple races	53 (10.6)	Conflict between races (eg, historical racism, double standards, “human race,” or colorblindness)
No specific race mentioned	56 (11.2)	Racism, antiracism, US politics, and political commentary

^a^Sample sizes for each race and ethnicity category are based on the total number of relevant Facebook posts coded from 2019 to 2023 (Table 1). Not relevant posts accounted for 19.8% (99/500) of the total posts.

To further illustrate how these themes were expressed in the data, The following sections provide representative examples of posts aligned with the 4 major themes: cultural pride, racism and stereotypes, antiracism, and US politics.

Textbox 2 highlights posts expressing cultural pride, often through celebration of heritage, historical milestones, or representation in national media.

Textbox 3 displays posts reflecting racism and stereotypes, including explicit or coded language that reinforces bias or discrimination, often masked in humor.

By contrast, Textbox 4 contains examples of posts incorporating antiracism, as evidenced by their condemnation of racist ideologies and views on inequitable treatment.

Finally, Textbox 5 contains examples of posts referring to various issues related to US politics, including commentary on elections, immigration policy, racial justice, and political figures.

**Examples of cultural pride memes in Facebook posts (2019-2023).**

- This victory is special for Hideki.

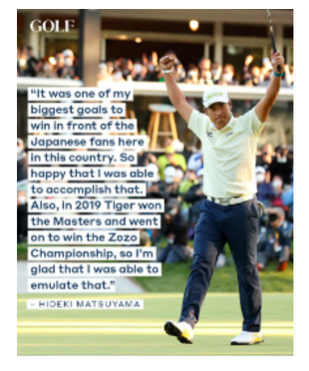

- Congratulations to the Honorable Ketanji Brown Jackson who was just confirmed to the Supreme Court of the United States in a 53-47 Senate vote! Once she’s sworn in, Justice Jackson will become the first Black woman on the highest court in the nation in its 232 year history. There have been 115 Justices in this country. 108 white men, 5 white women, and 2 Black men. I’m thrilled to witness this moment. 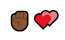 #BlackGirlMagic #SupremeCourtJustice #Herstory #KetanjiBrownJackson #BlackFactsxARYE

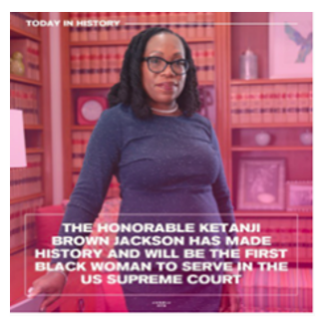

- Cuban American artist Celia Cruz will be the first Latina singer featured as part of the American Women Quarters Program, the US Mint announced. Read more: https://cnn.it/3YwPaDX:=:https://www.cnn.com/style/article/celia-cruz-us-quarter/index.html

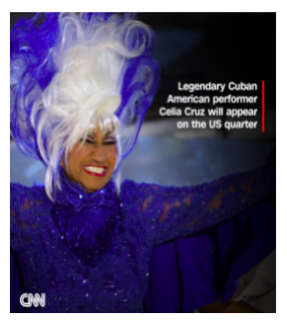

- Next weekend, Cherokee Nation will join the Eastern Band of Cherokee Indians and the United Keetoowah Band of Cherokee Indians to share the story of the Cherokee people. The tribes host Cherokee Days at the Smithsonian’s National Museum of the American Indian in Washington, DC. For more information, visit CherokeeDays.com.

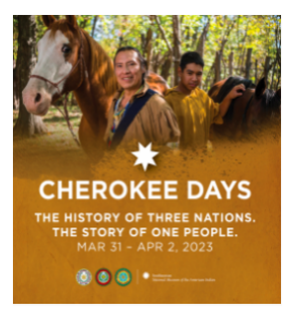

**Examples of racism and stereotypes memes in Facebook posts (2019-2023).**

- Life hacks 
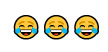

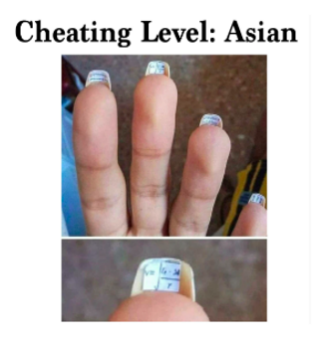

- Southern Breeze

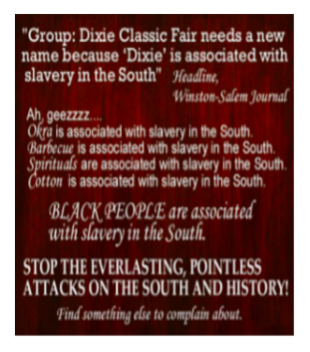

- Poll: Many Americans Know Illegals Create Risk of Mass Casualty Internal Attack on US Ranging from 9/11 Style To A More Systematic Nationwide Uprising

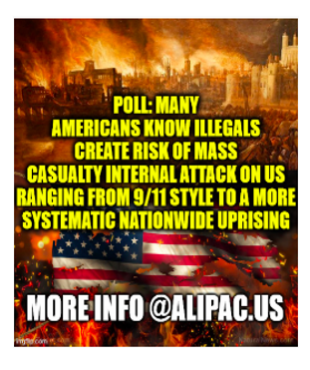

- Tik tok is wild...you just open the app and wham someone is lecturing you on a random non issue they made up in their head while combing a double ended dildo through their hair edit: the comment section is a dumpster fire and thats specificly because tiktok posts are curated to be beacons for anger dumping. thats the whole app meta concept.

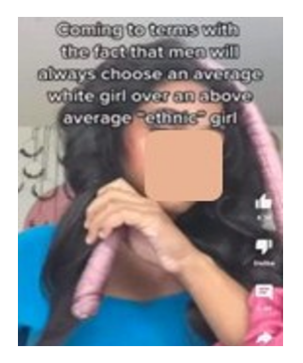

**Examples of antiracism memes in Facebook posts (2019-2023).**

- The two failings America has that are at the core of our destruction are racism and misogyny. If we have a movement that exposes how wrong that is and how oneness can save US as a society our world would be a much better place. Start by rejecting those bigotries by VOTING out anyone who espouses those beliefs. 2020

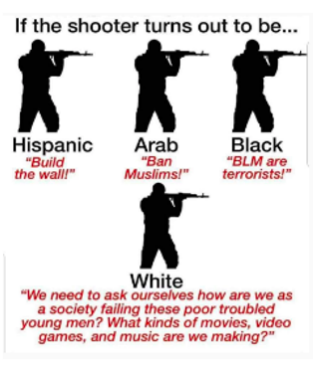

- Grow 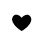 [antiracism: Fixed to Growth Mindset Fixed-Comfort: “I don’t know where to start or what to say.” Growth-Courage: “First I will listen/read/watch. I will speak against injustice.” Fixed-Comfort: “I don’t want to get it wrong or get called out.” Growth-Courage: “I will make mistakes, no doubt about it. I will be grateful for the lesson.” Fixed-Comfort: “It won’t make a difference what I do. Nothing is going to change.” Growth-Courage: “Things happen when I take risks and become part of something bigger.” Fixed-Comfort: “I don’t get involved in politics. I don’t have time.” Growth-Courage: “This is a human rights issue. This matters. I will make time.”]

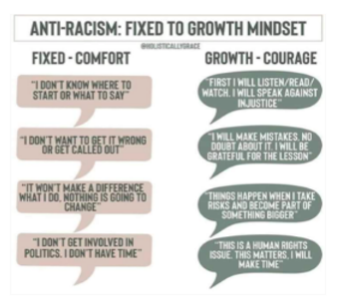

- Your belief that you haven’t personally benefited from white privilege doesn’t mean white privilege doesn't exist. (What people mostly mean when they make statements like “white privilege never helped me!” Is that they weren’t economically well off enough to benefit from class privilege, too.;

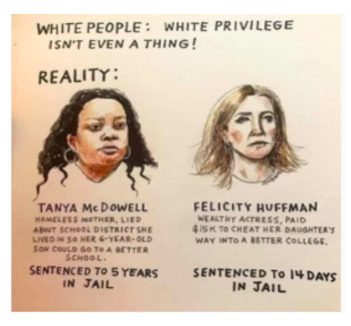

- None of us are free, until all of us are free.

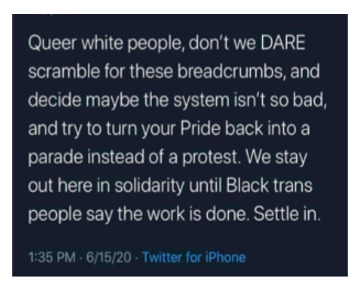

**Examples of US politics memes in Facebook posts (2019-2023).**

- Truth

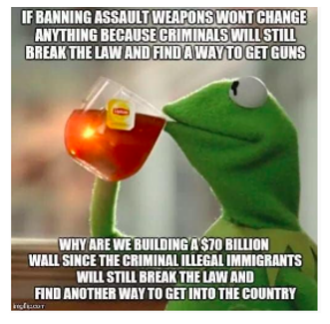

- [Chris]

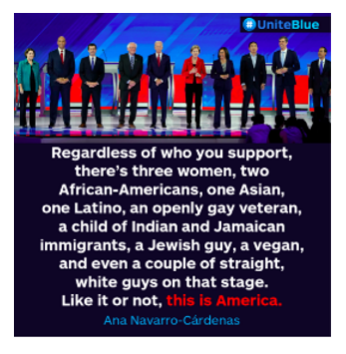

- Shameful and disgusting.

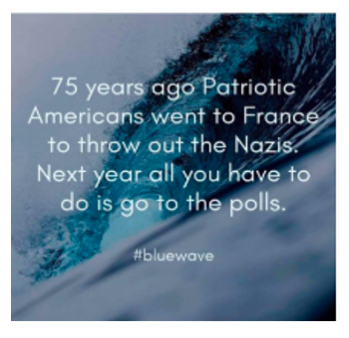

- #BREAKING — Four Proud Boys members found guilty of seditious conspiracy in Jan. 6 trial — https://nbcnews.to/42rUoCu:=:https://www.nbcnews.com/politics/justice-department/jury-reaches-verdict-proud-boys-seditious-conspiracy-trial-rcna81129 This is #BreakingNews worthy.

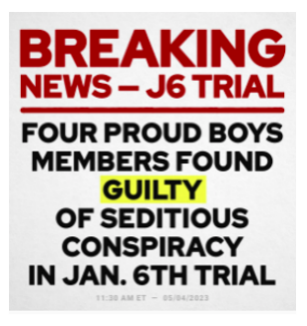

## Discussion

### Principal Findings

The rapid advancement of technology has profoundly reshaped social media platforms and the features they offer, creating a dynamic and constantly evolving landscape. Communication on these platforms is increasingly multimodal, incorporating various models such as text, images, and videos, which not only enhances the richness of content but also increases its interpretive complexity. This evolution necessitates a more intricate and critical understanding of how to engage with online content beyond traditional text-based evaluations, enabling a more nuanced interpretation of cultural and sentiment trends.

In this study, we qualitatively explored Facebook posts related to race and ethnicity, highlighting how images provide a cultural context and subtle cues that text alone cannot convey. This study advances previous literature by explicitly integrating multimodal analysis, which reveals complex, layered meanings in racial discourse often overlooked in text-only studies. For example, the nuanced use of humor and sarcasm in images demonstrates how stereotypes can be both masked and reinforced in ways that complicate interpretation. Furthermore, this work highlights a sustained expression of cultural pride across multiple racial and ethnic groups outside of crisis moments. This finding broadens the understanding of how identity affirmation operates continuously on social media, rather than solely during episodic activism. Finally, by mapping thematic content across a 5-year span and multiple groups, the study captures the interplay between identity-specific concerns and wider societal events shaping discourse on Facebook instead of examining one point in time.

### Chronology of Posts and Germane Current Events

The research team described qualitative content themes in Facebook posts regarding race and ethnicity, providing an overview of the landscape of image-based content creation and engagement. The qualitative content themes that were highly represented throughout the sample and across multiple race and ethnicity categories included US politics, cultural pride, racism, and antiracism. Approximately 19.8% (99/500) of the study sample was not relevant as they did not contain explicit or allusions to any race- and ethnicity-related information based on the research team’s ability to analyze the post. These posts may have appeared in the sample due to words having different meanings based on the context of the posts, such as sanctuary in terms of immigration versus animal sanctuaries.

The years 2019 to 2023 were an important time period for race-related discussions. Some of the most consequential moments include national discussions of immigration (eg, Trump’s administration immigration policies), the killing of George Floyd and a rise in the Black Lives Matter movement, increased racism against Asians being blamed for the COVID-19 pandemic, and sharp racial health disparities in the pandemic outcomes, among other cultural events that occurred [28-32]. The increase in mentions of Arab, Middle Eastern, and Muslim-related terms in 2021 may have been associated with the 20th anniversary of the September 11, 2001, terrorist attacks (commonly 9/11) in the United States [33].

### Cultural Pride

Social media can influence public perceptions on a variety of issues, and the tone of posts can reinforce or challenge common perspectives [34,35]. The findings of the study indicate that posts with a predominantly positive or neutral tone often included aspects of cultural pride (Textbox 2). Posts, such as the “Hideki” and the “Cuban American artist Celia Cruz” in Textbox 2, portray Hideki (of Japanese ancestry) and Cuban Americans as resourceful and talented. Studies have found that the use of a positive tone in posts elicits a positive response from social media users, who are more receptive to the message and likely to respond in a positive manner as a result [36]. Expression of cultural pride through image-based content can not only allow ethnic groups to denounce color-blind ideologies through racial silencing but can also foster a greater sense of self-esteem and competence among racial minoritized groups [5].

Previous research on social media found a similar theme regarding cultural pride. One study examining ethnic media found that ethnic social media use is often associated with increased ethnic pride and performance, unlike mainstream media use, which is associated with low self-esteem for ethnic users [37]. Other studies have found that individuals belonging to racially minoritized groups resist racial silencing through posts associated with cultural pride [5,38]. While these studies analyze user profiles, a study examining themes on #BlackLivesMatter tweets (regardless of user identity) found that while many posts expressed negative sentiments, posts with references to pride, optimism, and positive emotions were also prominent [39]. These results are similar to the study’s findings that positivity and cultural pride constituted a substantial portion of Facebook image-based content that contained terms related to race and ethnicity.

### Racism and Stereotypes

Often, in the absence of images, the text alone may appear neutral or benign. However, the addition of an image can dramatically alter the perspective of the text, bringing to light the true message and intention of a post. A common theme from the study found that Facebook posts perpetuate the stereotyping of racially minoritized groups. Results indicate that humor masking is often used to conceal hateful intentions and stereotyping, as also seen in other studies [18-20]. For instance, in Textbox 3, the phrase “life hacks” alone lacks interpretability without additional context. However, the inclusion of an image showing cheat sheets glued to fingernails, along with the image text “cheating level: Asian,” reveals a deeper meaning. The post subtly conceals and reinforces the typical “smart Asian” stereotype with humor, thus aiding the widespread distribution of such posts. Prior studies have also found, consistent to the findings in this study, that posts containing aspects of stereotyping or racism also tend to have neutral tones (as seen in the “poll: many Americans know...” post in Textbox 3), so that their opinionated content can be classified as “informational” or even as a positive stereotype [40]. Positive stereotypes, as in the “cheating level: Asian” post, attribute the success of racially minoritized groups to their racial and ethnic identity rather than their abilities and strengths [40].

Masked stereotyping can create false and negative perceptions of racially minoritized groups, as suggested by the racial formation theory [9,10]. According to cultivation theory, long-term exposure can distort users’ views of what topics relate to different races and their perception of others’ stances in relation to those topics, possibly working synergistically with social media’s undermining of the spiral of silence theory. In addition to the masking of racist or stereotypical posts, social media makes it easy to spread hateful messages through online communication (rather than in-person conversation) and the presence of like-minded online acquaintances, which creates a clustering of supporters of racist and stereotypical ideologies [41,42]. This both facilitates and normalizes racial microaggression on social media, which can create incorrect and hate-filled concepts of marginalized groups, as postulated by the racial formation theory [43].

Furthermore, this normalization perpetuates stress for targeted groups [3] due to the difference between how members of racially minoritized groups perceive their own identity and how that identity is perceived and expressed by others. In turn, the compounding negative effects on mental health could adversely affect physical health and social, economic, and political outcomes for marginalized groups, thus serving as a social determinant of health [6,7]. Therefore, it is necessary for social media users to be familiar with the different ways stereotypical and racist ideologies can manifest themselves through posts, especially if those ideologies and hateful intentions are masked in image-based content.

### Antiracism

Many antiracist posts on social media include a call to action, as can be seen in Textbox 4 (ie, the “fixed to growth mindset” and “none of us are free...” images) [44-46]. The call to action usually encourages the reader to decentralize authority and challenge implicit racism embedded in the status quo. Disseminating antiracist content via social media can provide a great opportunity to promote antiracism and influence real-life behavior on both interpersonal and intrapersonal levels. Interpersonally, people moved by social media posts are encouraged to oppose racism in everyday life [45]. Antiracism engagement on social media has positive mental health effects, such as decreased depression and anxiety, in Black, White, and Hispanic people [47]. Many antiracist posts use sarcasm and humor to convey their messages. So, while sarcasm is often used to mask racist ideology and vent negative emotions, it can also be used to promote antiracist ideals and have positive effects on mental health [13,48].

### Social Media and Political Views

Social media plays an important role in shaping a nation’s collective political will, or the will of a subgroup within a country [34]. For instance, disinformation often contributes to anti-immigrant racism. Neutral and informative content, such as the “breaking news” image in Textbox 5, cannot replace traditional media as a source of political knowledge. In its current state, social media reporting is inadequate to dispel disinformation [49]. Furthermore, social media frequently normalizes expressing anti-immigrant opinions not held by the majority, weakening the spiral of silence theory [8]. The normalization of anti-immigrant fervor also strengthens the cultivation theory, as users’ views of how others respond to issues, such as immigration, are distorted by long-term media exposure.

Once a consensus is reached through various means, many collective political wills make up the political landscape. Politicians gauge what views are popular and monitor their relations with the public using social media [50,51]. The posts captioned “[Chris]” and “shameful and disgusting” in Textbox 5 are examples of social media posts that represent user views of politicians and political parties. However, the political landscape and its constituent collective wills are ephemeral. Social media may initially draw attention to current events that encourage racism or antiracism, but interests can be transitory [52,53].

### Strengths and Limitations

This study offers several strengths that contribute to its overall rigor and relevance. First, the inclusion of both images and text, rather than relying on text alone, provided a more holistic view of how race and ethnicity were represented on Facebook. Second, all posts were double coded with an interrater agreement target of at least 80%, followed by consensus coding to achieve 100% alignment, ensuring a high level of reliability and consistency throughout the analysis. Finally, the 5-year time span of the dataset enabled the study to capture trends across key sociopolitical moments.

The study also had several limitations. The sample consisted of 100 posts per year, which allowed for manageable and detailed analysis, but a larger sample size may have revealed a wider range of perspectives and discourse patterns. With 500 posts, the sample size limited generalizability, particularly for specific race and ethnicity categories. For example, the “Indigenous or Native American” category included 10 (2%) posts, so readers should be cautioned against drawing strong conclusions from small subgroups. Facebook is known to overrepresent racially minoritized groups relative to their proportion of the US population. This representation may have influenced the content trends observed, but a larger sample might reveal different or additional thematic patterns.

To enhance representativeness, posts were randomly selected from a broad pool of publicly available Facebook content through CrowdTangle. It should be noted that the sample did not capture discussions from private groups and interpersonal conversations on Facebook. These other forums may have provided a more holistic perspective of the content. Another limitation is that Facebook may have removed very negative or harmful content before data collection. Even though these types of posts might have significant reach before deletion, they would not appear in the CrowdTangle dataset. Therefore, it is possible that the sample could have skewed more positive or affirming than the actual discourse.

Furthermore, the study relied on a keyword-based sampling strategy, which may have excluded posts with more implicit or nuanced references to race. To mitigate this, the research team developed the keyword list using both academic literature and a public database, and refined it during pilot testing. Even with an extensive keyword list, it is still possible that some posts addressing race and ethnicity did not include any of the specified keywords and were excluded. To strengthen the development of the keyword strategy and interpretation of data, the research team included members who identify as Asian, Black, and Hispanic.

### Conclusions

The results from the study highlight 2 key findings. Image-based posts and content on Facebook highlight positive ideas about racial and ethnic groups, such as cultural pride and antiracism; negative ideas, such as racism; and biased posts about US politics. This study demonstrated that it is more complicated to interpret image-based posts because of the subtle ways in which an image may reference race and ethnicity but does not explicitly mention it, or when there is a contradiction in the ideas portrayed in the image versus the text. That is, an image and its accompanying text can align in ideation, or a negative image could be accompanied by a positive text that opposes it or vice versa. Given that the findings of the study describe content themes regarding race and ethnicity, further research could examine themes in posts as related to other sociodemographic factors, such as gender identity, sexual orientation, age, socioeconomic status, and intersecting identities.

Future research could also expand the scope of this study by conducting a quantitative content analysis of image-based posts (including the prevalence of certain content themes) and by conducting a more thorough analysis of posts’ tone and sentiment. Subsequent studies may also find it beneficial to examine how social media users post about a specific race or ethnicity to better understand users’ attitudes or intentions toward a particular marginalized group. Finally, previous studies have found that identical posts are received differently in mainstream social media and social media subcultures or social media groups centered around common cultures [54]. However, how users post about sociodemographic factors in mainstream social media, in contrast to subgroups, is an unexplored terrain that future studies could explore. It would be valuable for future studies to expand the dataset using artificial intelligence tools to classify a larger volume of posts from platforms such as CrowdTangle. In conclusion, increasing our ability to decode posts related to race and ethnicity on Facebook can help inform research and intervention to boost the positive impacts and reduce the harmful effects of racism on social media.

## Appendix

Multimedia Appendix 1 Race terms used in keyword filtering.

## Data Availability

The datasets generated or analyzed during this study are available in the data repositories [22,23].
