# Supplementary material for: Race and Ethnicity in Facebook Images and Text: Thematic Analysis
Source: JMIR Form Res. 2025 Sep 4;9:e62713. doi: 10.2196/62713 (PMC12447004; doi:10.2196/62713)
Supplement: Multimedia Appendix 1 [file formative_v9i1e62713_app1.docx]

**eTable 1.** Race terms used in keyword filtering

| **Items** | **Race** |
| --- | --- |
| aapi | asian |
| abg's | asian |
| chinese | asian |
| afghans | asian |
| almond shaped eyes | asian |
| an abg | asian |
| asian | asian |
| asian indian | asian |
| asians | asian |
| aznbbygirl | asian |
| bamboo coon | asian |
| bangalees | asian |
| bangladeshi | asian |
| bangladeshi | asian |
| bengalis | asian |
| buddhahead | asian |
| burmese | asian |
| cambodian | asian |
| cambodians | asian |
| chiegro | asian |
| chinaman | asian |
| asian | asian |
| asian | asian |
| asians | asian |
| ching chong | asian |
| ching-chong | asian |
| chink | asian |
| chinks | asian |
| chinky | asian |
| chonky | asian |
| coconut nigger | asian |
| coolie | asian |
| cracker jap | asian |
| cunt-eyed | asian |
| dink | asian |
| dog muncher | asian |
| dog-muncher | asian |
| dothead | asian |
| east asian | asian |
| filipino | asian |
| filipinos | asian |
| fingernail rancher | asian |
| fresh off | asian |
| ganesha | asian |
| gook | asian |
| gookaniese | asian |
| gookemon | asian |
| gooky | asian |
| gyppo | asian |
| hawaiianculture | asian |
| hindi | asian |
| hindu | asian |
| hinduism | asian |
| hindus | asian |
| hindutva | asian |
| huns | asian |
| indonesian | asian |
| jap | asian |
| japs | asian |
| japanese | asian |
| kashmiri | asian |
| kashmiris | asian |
| korean | asian |
| koreans | asian |
| koreanstyle | asian |
| koreas | asian |
| lao | asian |
| laotian | asian |
| ling ling | asian |
| little hiroshima | asian |
| malayali | asian |
| malaysian | asian |
| maori | asian |
| mongol | asian |
| mongolian | asian |
| mongolians | asian |
| mongols | asian |
| nepal | asian |
| nepalese | asian |
| noodle nigger | asian |
| north korean | asian |
| oriental | asian |
| orientals | asian |
| asian | asian |
| pajeet | asian |
| paki | asian |
| pashtuns | asian |
| philippine | asian |
| polynesian | asian |
| polynesians | asian |
| punjab | asian |
| pyongyang | asian |
| rice burner | asian |
| rice nigger | asian |
| rice rocket | asian |
| rice-nigger | asian |
| rohingya | asian |
| samoan | asian |
| sideways cooter | asian |
| sideways pussy | asian |
| sikhs | asian |
| slanted eye | asian |
| slant-eye | asian |
| slanty eye | asian |
| slanty eyed | asian |
| slopehead | asian |
| south asian | asian |
| squinty | asian |
| sumerians | asian |
| tagalog | asian |
| taiwanese | asian |
| tamil | asian |
| teaceremony | asian |
| thai | asian |
| thais | asian |
| thin eyed | asian |
| thineyed | asian |
| thin-eyed | asian |
| tibetan | asian |
| currymuncher | asian |
| uyghurs | asian |
| vietcong | asian |
| vietnamese | asian |
| zipperhead | asian |
| wuhanvirus | asian |
| wuhan virus | asian |
| china virus | asian |
| chinavirus | asian |
| asianvirus | asian |
| asian virus | asian |
| kungflu | asian |
| kung flu | asian |
| yellow peril | asian |
| commie cough | asian |
| wuhanic plague | asian |
| madcau disease | asian |
| chingchongvirus | asian |
| kungflufighting | asian |
| kungfuflu | asian |
| chingchongprague | asian |
| commiecough | asian |
| wuflu | asian |
| chinadisease | asian |
| prayforchina | asian |
| blamechina | asian |
| wuhanpneumonia | asian |
| itschinasfault | asian |
| chinacorona | asian |
| hindoo | asian |
| pacific islander | asian |
| chamorro | asian |
| guamanian | asian |
| #blacklivesmatter | black |
| #blktwiter | black |
| #tamirrice | black |
| african american | black |
| african americans | black |
| african't | black |
| africoon | black |
| afro caribbean | black |
| afro-caribbean | black |
| atatianajefferson | black |
| atatiana jefferson | black |
| backtheblue | black |
| banjo lip | black |
| bantuknots | black |
| biscuit lip | black |
| bix nood | black |
| black ppl | black |
| black people | black |
| black boy | black |
| black boys | black |
| black female | black |
| black girl | black |
| black girls | black |
| black history | black |
| black lives | black |
| black male | black |
| black man | black |
| black men | black |
| black panther | black |
| black twitter | black |
| black woman | black |
| black women | black |
| blackeconomics | black |
| blackexcellence | black |
| blackgirlmagic | black |
| blackgirls | black |
| blackisbeautiful | black |
| blackity | black |
| blacklivesmatter | black |
| blacklove | black |
| blackpeople | black |
| blackpride | black |
| blacks | black |
| blacktwitter | black |
| blackunity | black |
| blackwomen | black |
| blks | black |
| blktwiter | black |
| blktwitter | black |
| blm | black |
| blue lives | black |
| bluelivesmatter | black |
| bootlip | black |
| buffie | black |
| bumper lip | black |
| burnt cracker | black |
| burrhead | black |
| bush-boogie | black |
| carribean people | black |
| chain dragger | black |
| congo lip | black |
| congolese | black |
| coon | black |
| coonass | black |
| coon-ass | black |
| coontang | black |
| dark skin | black |
| darkskin | black |
| darkey | black |
| darkie | black |
| darky | black |
| drc | black |
| ebennettart | black |
| ericgarner | black |
| eric garner | black |
| ethiopian | black |
| ethiopians | black |
| field nigger | black |
| freddie gray | black |
| freddiegray | black |
| golliwog | black |
| groid | black |
| haitians | black |
| hotep | black |
| jamaican | black |
| jamaicans | black |
| jigarooni | black |
| jigga | black |
| jiggabo | black |
| jigger | black |
| jim crow | black |
| knuckle-dragger | black |
| koon | black |
| light skin | black |
| lightskin | black |
| mandinka | black |
| moulie | black |
| mud people | black |
| n word | black |
| negro | black |
| negroes | black |
| negros | black |
| nevisian | Black |
| nigette | black |
| nigga | black |
| niggah | black |
| niggas | black |
| nigger | black |
| niggers | black |
| nigglet | black |
| nigglets | black |
| niglet | black |
| nig-ngo | black |
| nsbe | black |
| n-word | black |
| pickaninny | black |
| policebrutality | black |
| porch monkey | black |
| sandrabland | black |
| sandra bland | black |
| sayhername | black |
| shopblack | black |
| slavery | black |
| slaves | black |
| somali | black |
| spearchucker | black |
| staywoke | black |
| take a knee | black |
| takeaknee | black |
| tamir + rice | black |
| tar baby | black |
| tar-baby | black |
| themelaninleague | black |
| africans | black |
| bahamas | black |
| bahamian | black |
| bahamians | black |
| bantu | black |
| blackcommunity | black |
| blackpanther | black |
| burundi | black |
| cameroon | black |
| caribbeans | black |
| congo | black |
| cuban | black |
| cubano | black |
| cubans | black |
| dominican | black |
| ghanaian | black |
| haitian | black |
| jigaboo | black |
| kenyan | black |
| liberian | black |
| mozambican | black |
| nigeria | black |
| nigerian | black |
| nigerians | black |
| niggress | black |
| nubians | black |
| rwandan people | black |
| shona | black |
| somalian | black |
| somalis | black |
| south african | black |
| sudan | black |
| sudanese | black |
| swahili | black |
| tanzania | black |
| tanzanian | black |
| usorelse | black |
| zambian | black |
| zimbabwean | black |
| new mexico border | exclude |
| beaner | hispanic |
| border bandit | hispanic |
| border control | hispanic |
| border fence | hispanic |
| border hopper | hispanic |
| border jumper | hispanic |
| border nigger | hispanic |
| border security | hispanic |
| border surveillance | hispanic |
| border wall | hispanic |
| boricua | hispanic |
| brazilians | hispanic |
| build a wall | hispanic |
| buildawall | hispanic |
| buildourwall | hispanic |
| buildthatwall | hispanic |
| buildthedamnwall | hispanic |
| buildthewall | hispanic |
| buildthewallnow | hispanic |
| central american | hispanic |
| chexican | hispanic |
| chicano | hispanic |
| chicanos | hispanic |
| chicanotattoo | hispanic |
| chicanx | hispanic |
| colombian | hispanic |
| colombians | hispanic |
| columbians | hispanic |
| costa rican | hispanic |
| dampback | hispanic |
| dayofthedead | hispanic |
| domican | hispanic |
| dominicans | hispanic |
| ecuadorian | hispanic |
| fence fairy | hispanic |
| fence hopper | hispanic |
| fence-hopper | hispanic |
| fesskin | hispanic |
| finishthewall | hispanic |
| growinguphispanic | hispanic |
| guatemalans | hispanic |
| hispandex | hispanic |
| hispanic | hispanic |
| hispanicheritagemonth | hispanic |
| hispanics | hispanic |
| hispano | hispanic |
| hondurans | hispanic |
| latina | hispanic |
| latinas | hispanic |
| latinbeauty | hispanic |
| latino | hispanic |
| latinos | hispanic |
| latins | hispanic |
| latinx | hispanic |
| latinxs | hispanic |
| mayans | hispanic |
| mex | hispanic |
| mexcrement | hispanic |
| mexican | hispanic |
| mexicancocina | hispanic |
| mexicanos | hispanic |
| mexicans | hispanic |
| mexican't | hispanic |
| mexico border | hispanic |
| mexicoborder | hispanic |
| mexicocity | hispanic |
| mexicoon | hispanic |
| mexihos | hispanic |
| nicaraguans | hispanic |
| panamanian | hispanic |
| peruvian | hispanic |
| puerto rican | hispanic |
| puerto ricans | hispanic |
| raza | hispanic |
| salsacolombiana | hispanic |
| salvadorans | hispanic |
| salvadoreans | hispanic |
| salvadorians | hispanic |
| secureourborder | hispanic |
| south american | hispanic |
| spic | hispanic |
| spick | hispanic |
| spig | hispanic |
| spigotty | hispanic |
| spik | hispanic |
| taco nigger | hispanic |
| tamaulipas | hispanic |
| thicklatina | hispanic |
| venezuelans | hispanic |
| wetback | hispanic |
| wetblack | hispanic |
| deport | immigrant |
| deportation | immigrant |
| deported | immigrant |
| deporting | immigrant |
| deports | immigrant |
| end sanctuary | immigrant |
| first generation immigrant | immigrant |
| foreigner | immigrant |
| foreigners | immigrant |
| go back where | immigrant |
| gobackwhere | immigrant |
| illegal alien | immigrant |
| illegal aliens | immigrant |
| illegal immigrant | immigrant |
| illegal immigrants | immigrant |
| illegalalien | immigrant |
| illegalaliens | immigrant |
| illegalimmigrants | immigrant |
| illegals | immigrant |
| imigrant | immigrant |
| imigration | immigrant |
| immagrant | immigrant |
| immagration | immigrant |
| immig | immigrant |
| immigrant | immigrant |
| immigrants | immigrant |
| immigrantion | immigrant |
| immigrants | immigrant |
| immigrates | immigrant |
| immigration | immigrant |
| immigrations | immigrant |
| immingrant | immigrant |
| migrant | immigrant |
| migrants | immigrant |
| naturalized | immigrant |
| openborders | immigrant |
| our country back | immigrant |
| ourcountryback | immigrant |
| sanctuary | immigrant |
| sanctuary cities | immigrant |
| sanctuary city | immigrant |
| sanctuarycities | immigrant |
| sanctuarycity | immigrant |
| sanctuarystate | immigrant |
| sanctuarystates | immigrant |
| second generation immigrant | immigrant |
| secure our border | immigrant |
| travel ban | immigrant |
| undocumented | immigrant |
| afganistan | middle eastern |
| afghanistan | middle eastern |
| afghanistani | middle eastern |
| afghanistans | middle eastern |
| andsf | middle eastern |
| arab | middle eastern |
| arabs | middle eastern |
| arabush | middle eastern |
| armenians | middle eastern |
| baghdad | middle eastern |
| ban islam | middle eastern |
| ban muslim | middle eastern |
| ban on muslims | middle eastern |
| banislam | middle eastern |
| banmuslim | middle eastern |
| banonmulsims | middle eastern |
| cairo coon | middle eastern |
| camel cowboy | middle eastern |
| camel fucker | middle eastern |
| camel jacker | middle eastern |
| camelfucker | middle eastern |
| camel-fucker | middle eastern |
| cameljacker | middle eastern |
| camel-jacker | middle eastern |
| canaanites | middle eastern |
| carpet pilot | middle eastern |
| carpetpilot | middle eastern |
| clit chopper | middle eastern |
| clit-chopper | middle eastern |
| clitless | middle eastern |
| clit-swiper | middle eastern |
| derka derka | middle eastern |
| derkaderka | middle eastern |
| diaper head | middle eastern |
| diaperhead | middle eastern |
| diaper-head | middle eastern |
| dune coon | middle eastern |
| dune nigger | middle eastern |
| dunecoon | middle eastern |
| dunenigger | middle eastern |
| durka durka | middle eastern |
| durka-durka | middle eastern |
| egyptians | middle eastern |
| freemiddle eastern | middle eastern |
| fuckmuslims | middle eastern |
| hambaya | middle eastern |
| hebrews | middle eastern |
| hijab | middle eastern |
| hijabi | middle eastern |
| hijabs | middle eastern |
| ilan | middle eastern |
| irani | middle eastern |
| iranian | middle eastern |
| irans | middle eastern |
| iraqs | middle eastern |
| irgc | middle eastern |
| islamist | middle eastern |
| islamists | middle eastern |
| islams | middle eastern |
| israels | middle eastern |
| jig-abdul | middle eastern |
| jihad | middle eastern |
| jihadi | middle eastern |
| jihadis | middle eastern |
| jihadist | middle eastern |
| jihads | middle eastern |
| kaaba | middle eastern |
| kafeir | middle eastern |
| kuffar | middle eastern |
| middle eastern | middle eastern |
| moroccan | middle eastern |
| moslem | middle eastern |
| mudshark | middle eastern |
| muhammadalijinnah | middle eastern |
| muslim | middle eastern |
| muslimban | middle eastern |
| muslin | middle eastern |
| muzrat | middle eastern |
| muzzie | middle eastern |
| operationpeacespring | middle eastern |
| pashtun | middle eastern |
| pegida | middle eastern |
| peshmerga | middle eastern |
| phoenicians | middle eastern |
| pisslam | middle eastern |
| qtip head | middle eastern |
| rag head | middle eastern |
| raghead | middle eastern |
| rapefugee | middle eastern |
| rug pilot | middle eastern |
| rug rider | middle eastern |
| rugpilot | middle eastern |
| sand flea | middle eastern |
| sand monkey | middle eastern |
| sand moolie | middle eastern |
| sand nigger | middle eastern |
| sand rat | middle eastern |
| sandflea | middle eastern |
| sandmonkey | middle eastern |
| sandmoolie | middle eastern |
| sandnigger | middle eastern |
| sandrat | middle eastern |
| saudis | middle eastern |
| shiptar | middle eastern |
| slurpee nigger | middle eastern |
| slurpeenigger | middle eastern |
| sun goblin | middle eastern |
| sunnis | middle eastern |
| syrias | middle eastern |
| tehran | middle eastern |
| turks | middle eastern |
| wahabi | middle eastern |
| whacky iraqi | middle eastern |
| whitegenocide | middle eastern |
| yemen | middle eastern |
| afg | middle eastern |
| afghan | middle eastern |
| al qaeda | middle eastern |
| al-qaeda | middle eastern |
| arabian | middle eastern |
| arabic | middle eastern |
| aramaic | middle eastern |
| asslifter | middle eastern |
| assyrian | middle eastern |
| badghis | middle eastern |
| baloch | middle eastern |
| bansharialaw | middle eastern |
| bhutanese | middle eastern |
| carpet kisser | middle eastern |
| death to islam | middle eastern |
| egyptian | middle eastern |
| farsi | middle eastern |
| goat fucker | middle eastern |
| haji | middle eastern |
| hajji | middle eastern |
| hamas | middle eastern |
| hojabi | middle eastern |
| impeachilhanomar | middle eastern |
| iranians | middle eastern |
| iraqi | middle eastern |
| iraqis | middle eastern |
| islam | middle eastern |
| islamic | middle eastern |
| islamics | middle eastern |
| israeli | middle eastern |
| israelis | middle eastern |
| isreali | middle eastern |
| jehad | middle eastern |
| jordanian | middle eastern |
| koranimal | middle eastern |
| kurd | middle eastern |
| kurdish | middle eastern |
| kurds | middle eastern |
| lebanese | middle eastern |
| lebanese | middle eastern |
| levantines | middle eastern |
| malayer | middle eastern |
| middle eastern | middle eastern |
| moosrats | middle eastern |
| moroccans | middle eastern |
| moslems | middle eastern |
| mud slum | middle eastern |
| mudslum | middle eastern |
| muslims | middle eastern |
| muzzies | middle eastern |
| muzzrats | middle eastern |
| middle eastern | middle eastern |
| palestinian | middle eastern |
| palestinians | middle eastern |
| persian | middle eastern |
| persian | middle eastern |
| persians | middle eastern |
| shias | middle eastern |
| syrian | middle eastern |
| syrianrefugee | middle eastern |
| syrians | middle eastern |
| taliban | middle eastern |
| taliban | middle eastern |
| tunisians | middle eastern |
| turkish | middle eastern |
| urdu | middle eastern |
| yazidi | middle eastern |
| yemeni | middle eastern |
| yiddish | middle eastern |
| bipoc | minority |
| brown people | minority |
| brownskin | minority |
| brown skin | minority |
| ethnics | minority |
| minorities | minority |
| myhousemyamerica | minority |
| people of color | minority |
| person of color | minority |
| poc | minority |
| pocs | minority |
| race traitor | minority |
| racism | minority |
| racist | minority |
| racists | minority |
| woc | minority |
| wog | minority |
| beaner shnitzel | multi-race |
| beanershnitzel | multi-race |
| biracial | multi-race |
| caublasian | multi-race |
| half breed | multi-race |
| half cast | multi-race |
| half-breed | multi-race |
| half-cast | multi-race |
| interracial | multi-race |
| intraracially | multi-race |
| bow bender | native american |
| buffalo jockey | native american |
| bushnigger | native american |
| cherry nigger | native american |
| hatchet-packer | native american |
| prairie nigger | native american |
| red nigger | native american |
| river nigger | native american |
| rivernigger | native american |
| squaw | native american |
| teepee creeper | native american |
| tee-pee creeper | native american |
| timber nigger | native american |
| timbernigger | native american |
| tomahawk chucker | native american |
| tomahawk-chucker | native american |
| tomahonky | native american |
| american indian | native american |
| apache | native american |
| apache indian | native american |
| apache nation | native american |
| apache tribe | native american |
| cherokee | native american |
| cherokee indian | native american |
| cherokee nation | native american |
| cherokee tribe | native american |
| chippewa indian | native american |
| chippewa nation | native american |
| chippewa tribe | native american |
| choctaw | native american |
| choctaw indian | native american |
| choctaw nation | native american |
| choctaw tribe | native american |
| hula | native american |
| iroquois indian | native american |
| iroquois nation | native american |
| iroquois tribe | native american |
| luau | native american |
| native american | native american |
| native americans | native american |
| native hawaiian | native american |
| navajo | native american |
| navajostrong | native american |
| navajoweavingrules | native american |
| pueblo indians | native american |
| pueblo nation | native american |
| pueblo tribe | native american |
| sioux | native american |
| sioux indian | native american |
| sioux nation | native american |
| sioux tribe | native american |
| tohono | native american |
| aid refugee | refugee |
| asylee | refugee |
| help refugee | refugee |
| migrant protection program | refugee |
| norefugeeban | refugee |
| refugee | refugee |
| refugeelivesmatter | refugee |
| refugee | refugee |
| refuges | refugee |
| resettlement | refugee |
| unhcr | refugee |
| we welcome refugee | refugee |
| welcome refugee | refugee |
| welcomerefugee | refugee |
| Jew | jewish |
| Soros | jewish |
| Zionism | jewish |
| Crypto-jew | jewish |
| Shyster | jewish |
| Blood libel | jewish |
| Dual loyalty | jewish |
| Globalist | jewish |
| Goyim | jewish |
| Holocough | jewish |
| Holocaust denial | jewish |
| Holohoax | jewish |
| Jewish lobby | jewish |
| Jew down | jewish |
| Kosher tax | jewish |
| Not the real jews | jewish |
| Zionazi | jewish |
| Zionazis | jewish |
| Smirking merchant | jewish |
| Zionist occupied government¬† | jewish |
| ZOG | jewish |
| Feuj | jewish |
| Jewsdontcount | jewish |
| Ziojew | jewish |
| Jewtard | jewish |
| Jewbag | jewish |
| UnBonJuif | jewish |
| SiJetaisNazi | jewish |
| Jewishprivileg¬† | jewish |
| Swastika | jewish |
| ashkenazim | jewish |
| cohencidence | jewish |
| jewish | jewish |
| judaism | jewish |
| kike | jewish |
| kippahs | jewish |
| kyke | jewish |
| loxism | jewish |
| stopzionism | jewish |
| zionist | jewish |
| zionists | jewish |
| jews | jewish |
|  |  |
